# The infected hematometra in a rudimentary noncommunicating horn misdiagnosed as pelvic mass: A case report

**DOI:** 10.1016/j.ijscr.2020.01.052

**Published:** 2020-02-11

**Authors:** Michaela Behrens, Michael Licata, Ji-Young Lee

**Affiliations:** a Department of Obstetrics and Gynecology, Sisters of Charity Hospital, University at Buffalo, Buffalo, New York, 14214, USA; b Department of Radiology, Sisters of Charity Hospital, University at Buffalo, Buffalo, New York, 14214, USA

**Keywords:** Hematometra, Non-communicating, Rudimentary horn, Müllerian anomaly, Case report

## Abstract

**Introduction:**

The rudimentary noncommunicating horn with a functional endometrial cavity is rare and often challenging to diagnose because of the variety in clinical features. We present a case of a patient for whom the diagnosis of a uterine horn was missed during the prior cesarean section, which later successfully treated with robotic-assisted laparoscopic removal of a rudimentary noncommunicating horn of uterus and ipsilateral tube.

**Presentation of case:**

A 20-year old woman, gravida 3 para 2, presented with a complaint of acute and severe pelvic pain with fever. Multiple imaging modalities of pelvis and abdomen showed an 8 cm right-sided pelvic mass with a tubular structure adjacent to the uterus. The pelvic inflammatory disease was diagnosed and treated with intravenous antibiotics. After reviewing multiple radiology images, Müllerian anomaly was suspected, and the rudimentary horn with the fallopian tube was confirmed via diagnostic hysteroscopy and laparoscopy. Subsequently, robotic-assisted laparoscopic removal of the right horn with the fallopian tube was performed.

**Discussion:**

Assessment of a rudimentary noncommunicating horn with unicornuate uterus can be achieved by several radiology methods, including computed tomography, magnetic resonance imaging, two and 3-dimensional ultrasonography, hysterosalpingogram, and sonohysterography. In addition, evaluation of concomitant skeletal and renal anomalies is essential in enhancing diagnostic accuracy. In our case, the Müllerian anomaly with delayed onset complications was diagnosed by multiple imaging studies and treated successfully.

**Conclusion:**

The early and correct diagnosis of the Müllerian anomaly remains difficult but essential as misdiagnosis can be associated with serious complications in patients.

## Highlights

• The rudimentary noncommunicating horn with a functional endometrial cavity is should be treated promptly to prevent obstetric and gynecologic complications.
• The MRI is essential in assessing Müllerian anomaly.
• The presence of concomitant congenital malformation, renal and skeletal anomalies, should raise the high suspicion of Müllerian anomaly.

## 1 Introduction

Congenital abnormalities of the Müllerian ducts are relatively common and contribute to complications in obstetrics and gynecology. A unicornuate uterus is caused by a failure of development in one Müllerian duct and frequently present with a rudimentary horn [1]. Most rudimentary horns are asymptomatic when the endometrium is both non-functional and noncommunicating [2]. However, rudimentary horns with a functional endometrial cavity can cause chronic pain, dysmenorrhea, and endometriosis, and they are often difficult to diagnose by noninvasive means [3]. Therefore, proper preoperative diagnosis and surgical treatment are essential to prevent serious complications in the future as well as to provide symptomatic relief to patients. We present the case of a patient for whom the diagnosis of a uterine horn was missed during the prior cesarean section, and we successfully treated with robotic-assisted laparoscopic removal of a rudimentary noncommunicating horn and ipsilateral tube causing severe pelvic pain. The case report described here is in line with the SCARE criteria [4].

## 2 Presentation of case

A 20-year-old woman, gravida 3 para 2, presented to the emergency department with acute and severe pelvic pain with fever 38.3 °C (101 °F). Her gynecologic and obstetrical history consisted of one cesarean section due to preterm prelabour rupture of membranes and increased fetal risk at 35 week-gestation in 2011. Her second pregnancy in 2013 was a successful vaginal birth after the cesarean section at 37 week-gestation. Then, the patient was treated for chlamydia infection about three months after her last delivery. She endorsed cyclic pelvic pain, particularly at the time of her regular menses. A patient denied any significant medical and family history.

**Fig. 1:**
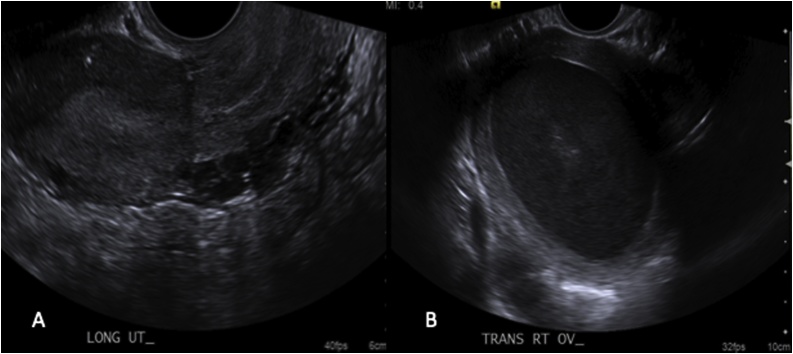
2D transvaginal Pelvic ultrasonography: A. Left unicornuate uterus, B. Hematometra in the non-communicating rudimentary horn was reported as right ovarian endometrioma.

**Fig. 2:**
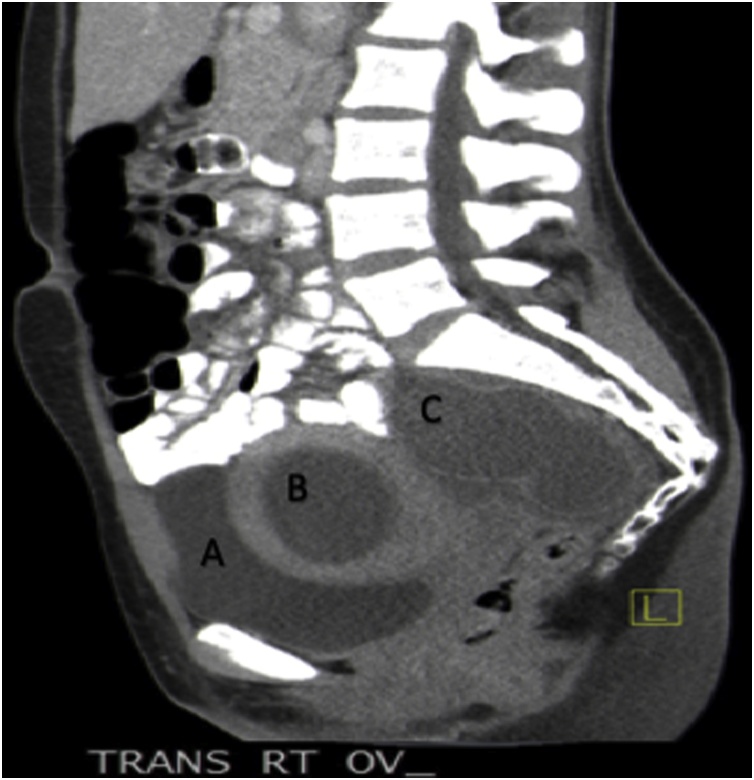
The sagittal plane of CT scan with oral contrast: A. Bladder, B. Hematometra in the non-communicating rudimentary horn with active cavity, misread as right ovarian complex mass, C. Right dilated fallopian tube.

**Fig. 3:**
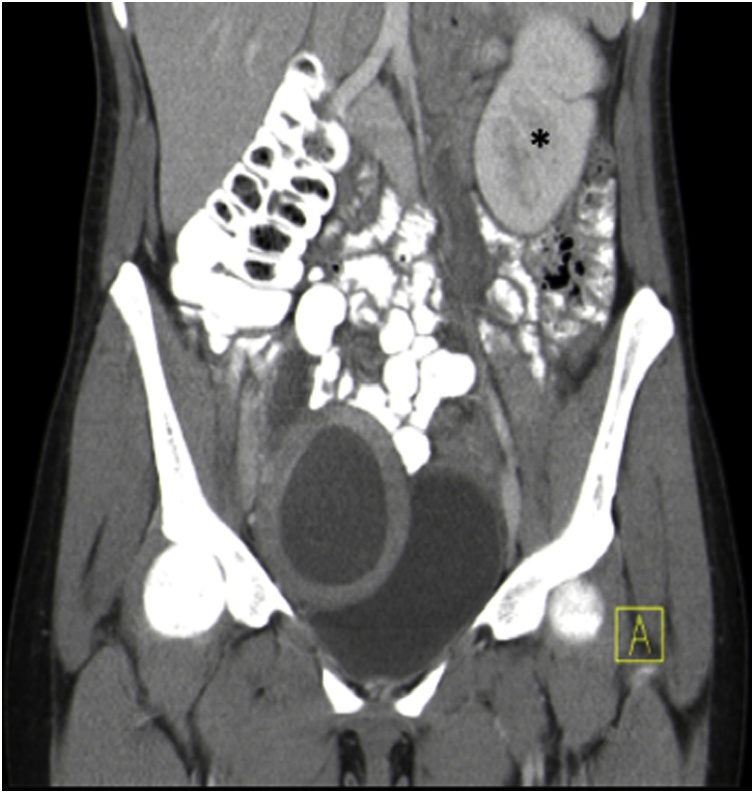
The coronary plane of CT scan with oral contrast: Note only one kidney on patient’s left side (*).

Upon arrival at the emergency department, her blood pressure was 119/76, heart rate of 74, respiratory rate of 16, and temperature of 38.3 °C. On the abdominal exam, there was tenderness in the right lower quadrant with the rebound but no rigidity or guarding. Her pelvic exam revealed purulent discharge coming from the cervical os and cervical motion tenderness on a bimanual exam. In the laboratory, her white blood cell count was 12,500/microliter, and hemoglobin was 12.4 g/dL. Her basic metabolic panel was found to be within normal limits. The ultrasound study was performed first and reported as right endometrioma of ovary measuring 8 cm in diameter (Fig. 1). The computed tomography (CT) of pelvis and abdomen was also read as a right enlarged adnexal mass measuring 8 cm in diameter with a dilated tubular structure adjacent to the mass (Fig. 2). In addition, the congenital absence of the right kidney was confirmed by the CT images (Fig. 3). The diagnosis of the tubo-ovarian abscess was made, and the patient was admitted and treated with intravenous antibiotics. The patient became afebrile and had improved symptoms, then discharged on hospital day 2 with seven-day course oral antibiotics.

**Fig. 4:**
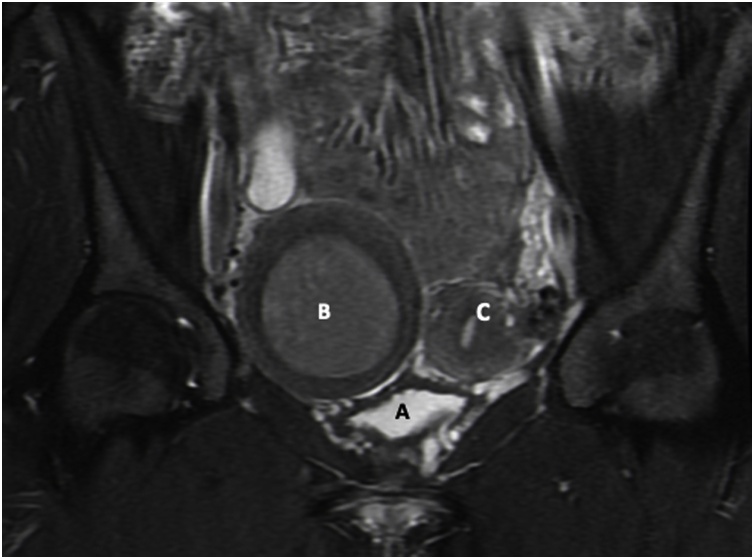
The coronary plane of MRI with IV contrast: A. Bladder, B. Hematometra in the rudimentary non-communicating horn with functioning cavity, misread as right ovarian cyst structure, C. Left unicornuate uterus.

The patient returned to the clinic for follow up in one week after being discharged from the hospital. Persistent pelvic pain was present on her right lower pelvic area. On further review of images with radiology department, the presence of concomitant renal anomaly, ipsilateral renal agenesis, raised a suspicion that the patient has Müllerian anomaly with complications, hematometra and associated hematosalpinx in a probable right uterine horn. Magnetic resonance image (MRI) of pelvis and abdomen was ordered to assess Müllerian anomaly. However, the MRI failed to classify the patient’s Müllerian anomaly and was read as a right enlarged pelvic mass, possible endometrioma of ovary, with displaced uterus to left pelvic area (Fig. 4).

The patient underwent an exam under anesthesia, diagnostic hysteroscopy as well as diagnostic laparoscopy. Hysteroscopic examination confirmed a single endometrial cavity with one cervix and vagina with no evidence of a septum and passage communicating with the right rudimentary horn. On laparoscopic exam, the patient was found to have a dilated and enlarged rudimentary right horn with normal uterus in her left side. The unicornuate uterus was noted to be normal in appearance with a normal tube and ovary. An enlarged and distended tubular structure was present adjacent to rudimentary horn and confirmed to be right fallopian tube with tubal obstruction. About three hundred mL of dark brown thick blood was removed from the right salpinx through salpingostomy. Subsequently, we performed a robotic-assisted laparoscopic removal of a right uterine horn as well as the right salpingectomy without any complication. The patient was discharged home the following day without complications and her recovery process was unremarkable. Two years later, the patient had a normal vaginal delivery of a term infant.

## 3 Discussion

A rudimentary horn with the unicornuate uterus results as a failure of the complete development of one of the Müllerian ducts and incomplete fusion with the contralateral side. In its classification of Müllerian anomalies, the American Fertility Society (AFS) categorizes a unicornuate uterus as class II, further divided into four subgroups. Group A includes a rudimentary horn with a cavity, communicating to the unicornuate uterus. Group B includes a rudimentary horn with a cavity, not communicating to the unicornuate uterus. Group C includes a rudimentary horn with no cavity. Finally, group D includes unicornuate uterus without a rudimentary horn [1]. This particular case would be considered “class II B,” as the cavity of the rudimentary horn was functioning and does not communicate with the uterus. Incidence of the unicornuate uterus accounts for 2.4–13% of these Müllerian anomalies. Rudimentary horns are found within 74% of these unicornuate uteri and classified as noncommunicating in the majority of cases [2].

Clinical presentations of a rudimentary horn are highly variable. A recent review by Jayasinghe et al. [3] studied 376 cases of rudimentary horns where, in many cases, 78% of patients were asymptomatic and did not present until the third decade of life. They often presented with progressive dysmenorrhea or abdominal pain after menarche thought to be caused by hematometra, hematosalpinx, and endometriosis. In our case, hematometra in a rudimentary horn was attributed to blocked fallopian tube after chlamydia infection. Women also can present with infertility as well as for pregnancy complications, including recurrent miscarriages, preterm labor, and malpresentation. Therefore, early diagnosis is essential, but early diagnosis remains difficult, as in our case. Our patient has a congenital absence of the right kidney, which raised suspicion of Müllerian anomaly. It is important to keep a high index suspicion for high-risk groups, especially those who have spinal, cloacal, or renal anomalies since they have an association of 45–60% with Müllerian anomalies [5].

Assessment of the rudimentary horn can be achieved by several methods, including MRI, two and three-dimensional ultrasonography, hysterosalpingogram, and sonohysterography. Jayasinghe et al. found the sensitivity of two-dimensional ultrasonography for diagnosis was only 26% [3]. However, it is the most common modality of investigation [2]. Our patient also had two-dimensional ultrasonography at first, but it failed to detect her Müllerian anomaly. Hematometra in a rudimentary horn along with ipsilateral hematosalpinx was interpreted as the endometrial cyst of the ovary in ultrasonography. Investigators have tried to improve the accuracy in diagnosing the complications associated with Müllerian anomalies. Tsafarir et al. created ultrasonographic diagnostic criteria in order to differentiate rudimentary horn pregnancies from tubal ectopic and bicornuate uterine pregnancies [6]. Buntugu et al. recently proposed a method involving placement of a catheter into the uterine cavity with a bulb inflated followed by a subsequent abdominal ultrasound with a full bladder [7]. Another recent study by Ghi et al. found a 3-dimensional (3D) transvaginal ultrasound to be a very accurate uterine anomaly diagnosis with a sensitivity of 93% and a specificity of 100% [8]. The advantages of this method include the ability to measuring distances and volumes of organs as well as navigation through a single plane while watching the effect on the other two. Advocates of the 3D ultrasound also claim ease of obtaining a coronal view with the entire endometrial canal, the relationship of the endometrium to the myometrium, and the uterine serosa. The MRI, however, remains as the gold standard, which has shown an accuracy of 96%. 3D-CTs have recently been utilized as well and offer a less-expensive alternative [9]. Despite multiple radiology image studies, the patient’s Müllerian anomaly was not well defined prior to the procedure. Considering the complexity of the surgical procedure and complications as a result, the diagnostic accuracy in the imaging studies is paramount in evaluating the size of the horn and communication between the horn and unicornuate uterus. The diagnostic criteria in radiology studies of Müllerian anomaly needs to be established in order to improve the outcome of surgical procedures.

Several cases of laparoscopic removal of noncommunicating uterine horns have been reported, and this appears to be the preferred approach. A descriptive study by Fedele et al. demonstrated the safety and effectiveness of laparoscopic removal of rudimentary noncommunicating horns [9]. Laparoscopic approaches seem to have comparable outcomes to laparotomy, but carry the benefits of minimally invasive surgery, including shorter hospital stay and shorter recovery along with better cosmetic outcomes. When an attachment is broad, the procedure is understandably more complex, and coagulation of the contributing arteries, including the ipsilateral uterine and the myometrial arcuate arteries of the contralateral uterine arteries, need to be carefully done. They also mentioned that the ureter ipsilateral to the rudimentary horn often has a higher course. The ipsilateral fallopian tube should always be removed to prevent a tubal pregnancy [10]. In our case, the rudimentary noncommunicating horn along with right fallopian tube was removed without any complications. There was no evidence of aberrant vessels or ureter around the rudimentary horn at the time of the procedure.

## 4 Conclusion

In conclusion, this case demonstrates the importance of early detection and prompt treatment of Müllerian anomaly to prevent any obstetrical and gynecologic complications. Given the difficulty in diagnosing and classifying Müllerian anomalies, it is important to keep a high index of suspicion for high risks groups, especially females with spinal, cloacal, or renal anomalies, and cautiously review the images of radiology studies. As a treatment option, Laparoscopic or Robotic assisted removal of rudimentary noncommunicating horn are feasible and preferred, compared with open procedure.

## Sources of funding

This research did not receive any specific grant from funding agencies in the public, commercial, or not-for-profit sectors.

## Ethical approval

Ethical approval was exempt from Catholic Health Institutional Review Board. Written informed consent for the report being submitted for publication has been obtained by the patient and is available upon request from the corresponding author.

## Consent

Written informed consent was obtained from the patient for publication of this case report and accompanying images. A copy of the written consent is available for review by the Editor-in-Chief of this journal on request.

## Author contribution

**Michaela Behrens:** Writing – Primary author, Original draft preparation, Visualization.

**Michael Licata:** Resources – Provided images of radiology studies.

**Ji-Young Lee:** Corresponding author, Patient’s gynecologist and performed the procedure, Writing- Review & Editing, Supervision.

## Registration of research studies

This is not applicable due to the nature of this case study.

## Guarantor

Ji Young Lee, MD, MPH, FACOG.

## Provenance and peer review

Not commissioned, externally peer-reviewed.

## Declaration of Competing Interest

The authors have no conflicts of interest to declare.
